# Validation of Effective Extracellular Vesicles Isolation Methods Adapted to Field Studies in Malaria Endemic Regions

**DOI:** 10.3389/fcell.2022.812244

**Published:** 2022-05-16

**Authors:** Matteo Zoia, Bibin Yesodha Subramanian, Klara Kristin Eriksson, Meera Sruthi Ravi, Shekoofeh Yaghmaei, Isabelle Fellay, Brigitte Scolari, Michael Walch, Pierre-Yves Mantel

**Affiliations:** ^1^ Faculty of Science and Medicine, Department of Oncology, Microbiology and Immunology, Anatomy Unit, University of Fribourg, Fribourg, Switzerland; ^2^ Department for BioMedical Research (DBMR), University of Bern, Bern, Switzerland

**Keywords:** malaria, Plasmodium falciparum, extracellular vesicles, microvesicles, salting-out, polyethylene glycol precipitation

## Abstract

Malaria affects the poorer regions of the world and is of tremendous health and economic burden for developing countries. Extracellular vesicles (EVs) are small vesicles released by almost any cells in the human body, including malaria infected red blood cells. Recent evidence shows that EVs might contribute to the pathogenesis of malaria. In addition, EVs hold considerable value in biomarker discovery. However, there are still significant gaps in our understanding of EV biology. So far most of our knowledge about EVs in malaria comes from *in vitro* work. More field studies are required to gain insight into their contribution to the disease and pathogenesis under physiological conditions. However, to perform research on EVs in low-income regions might be challenging due to the lack of appropriate equipment to isolate EVs. Therefore, there is a need to develop and validate EV extraction protocols applicable to poorly equipped laboratories. We established and validated two protocols for EV isolation from cell culture supernatants, rodent and human plasma. We compared polyethylene glycol (PEG) and salting out (SA) with sodium acetate for precipitation of EVs. We then characterized the EVs by Transmission Electron Microscopy (TEM), Western Blot, Size-exclusion chromatography (SEC), bead-based flow cytometry and protein quantification. Both protocols resulted in efficient purification of EVs without the need of expensive material or ultracentrifugation. Furthermore, the procedure is easily scalable to work with large and small sample volumes. Here, we propose that both of our approaches can be used in resource limited countries, therefore further helping to close the gap in knowledge of EVs during malaria.

## Introduction

Malaria remains one of the greatest life-threatening diseases worldwide. It is caused by several Plasmodium species of parasites that are introduced into the bloodstream *via* mosquito bites. Mostly at risk are populations in tropical and subtropical areas of over 100 countries (WHO, 2020). The severe disease develops during the blood stage, when the parasite replicates inside red blood cells (RBCs). Several factors derived from both the parasites and host determine the severity and outcome of the disease (Miller et al., 2002; Schofield and Grau, 2005; Coban et al., 2018). Recently, extracellular vesicles (EVs) have been described to contribute to the pathological processes during malaria infection, and in particular during cerebral malaria and severe anemia (Combes et al., 2005; Coltel et al., 2006). The earliest reports focused on the EVs released by endothelial cells, lymphocytes and platelets (Combes et al., 2004). In fact, endothelial cell derived EVs were elevated in a population of children infected with *P. falciparum*, the highest level was observed at admission in the group of children suffering from cerebral malaria (Combes et al., 2004). In addition to the EVs derived from host, more recent evidence suggests that EVs secreted by Plasmodium infected red blood cells (iRBCs) might contribute to the development of the disease as well (Antwi-Baffour et al., 2020). EVs are an heterogenous collections of vesicles characterized by differences in sizes and biogenesis pathways (Valadi et al., 2007; Babatunde et al., 2018; Théry et al., 2018). EVs are involved in many biological processes, including cellular differentiation and immune regulation. Despite the differences between each biogenesis pathway, a shared characteristic of all EVs released is that their membrane composition reflects their cellular origin. Since EVs contain molecules derived from their mother cells, they constitute a promising source of biomarkers readily available in biofluids.

In malaria, iRBC derived EVs were shown to mediate the transfer of DNA between parasites and to promote the differentiation from the asexual parasites towards gametocytes, to initiate the transmission stage from the human to the mosquito host (Mantel et al., 2013; Regev-Rudzki et al., 2013; Sampaio et al., 2017). Therefore, it seems that the parasites have developed strategies to synchronize and coordinate their behavior during infection. In addition, to their role in parasite-parasite communication, EVs have potent immunoregulatory properties. For instance, EVs can stimulate monocytes and macrophages to secrete proinflammatory cytokines and chemokines (Couper et al., 2010; Sisquella et al., 2017; Mbagwu et al., 2019; Ofir-Birin et al., 2021). EVs secreted by mast cells worsen the development of cerebral malaria in the rodent malaria (Huang et al., 2021). EVs produced during *P. vivax* infections are taken up by human spleen fibroblast, in which they induced the expression of ICAM-1 to potentiate parasite sequestration (Toda et al., 2020). Furthermore EVs might be involved in drug resistance development against some antimalaria drugs (Tandoh et al., 2021). Beside their role in the development of the disease EVs might be used as vaccine to prevent the development of severe malaria (Martin-Jaular et al., 2011) and can be used a drug delivery tools against parasitic disease (Borgheti-Cardoso et al., 2020). It has been reported that during malaria infection by *P. falciparum* or *P. vivax*, the concentration of EVs increases in the blood of malaria patients (Campos et al., 2010; Pankoui Mfonkeu et al., 2010; Antwi-Baffour et al., 2020). Interestingly EVs might be used as biomarkers to detect liver infections in patients infected with *P. vivax* (Gualdrón-López et al., 2018). Although evidence of a prominent role of EVs during malaria is growing, a complete understanding of their physiological function and relevance is still lacking. In fact, most of the knowledge is coming from *in vitro* experiments, more *in vivo* data and field trials are required to fully understand EVs biology and contribution to the pathogenesis of malaria. Despite progresses in EV research, it remains a challenge to purify biologically intact EVs from biofluid samples of limited volumes (Babatunde et al., 2020).

Differential centrifugation remains the gold standard approach to enrich and purify EVs (Momen-Heravi et al., 2013). The separation relies on the differences in the sedimentation speed between EVs and other particles. The first centrifugation steps are meant to remove cellular debris and impurities. Finally, EVs are collected by ultracentrifugation at 100′000 g, followed in some protocols by density gradient, resulting in enhanced sample purity. In fact, iso-osmotic gradients such as sucrose allow to separate vesicles based on their buoyant density, therefore eliminating proteo-lipid complexes (Théry et al., 2006). Size-exclusion chromatography allows to separate molecules varying in their hydrodynamic radius by passing them through a column containing a porous gel. While small molecules are retained in the pores, the larger molecules such as EVs migrate faster through the matrix and are eluted first (Takov et al., 2019). Affinity immunocapturing is an interesting alternative, it is based on the presence of known, specific markers on the surface of the EVs. Therefore, antibodies can be used to target and bind those receptors. Antibodies can be coupled to magnetic beads to allow the EV capture by using a magnet. Therefore, affinity immunocapturing outer perform other methods in terms of specificity as most contaminants are removed (Nakai et al., 2016). However, the relatively low expression of the receptor on the EVs may also reduce the isolation yield.

In addition, several commercial kits are available, however their high cost limits the usage for large volumes of cell culture supernatants or large number of clinical samples, particularly in resource limited regions. Thus, a simple, inexpensive and rapid EV isolation method that can process cell culture media or diverse biofluids is an essential but unmet need in many research and clinical settings.

Recently, simpler and cheaper approaches such as polyethylene glycol (PEG) precipitation and salting out have been described. Here, we analyzed the suitability of those two methods to extract RBC derived EVs derived from *in vitro* and *in vivo* samples. We first demonstrate that PEG or salting out based precipitation can efficiently isolate intact RBC EVs from cell culture media. We then show that we can purify EVs from mouse and human plasma. In conclusion, both approaches efficiently purified EVs. Our platform has a broad application to the processing of EVs in malaria research.

## Results

### Overall Strategy to Compare Side By Side Extraction of EVs

**FIGURE 1:**
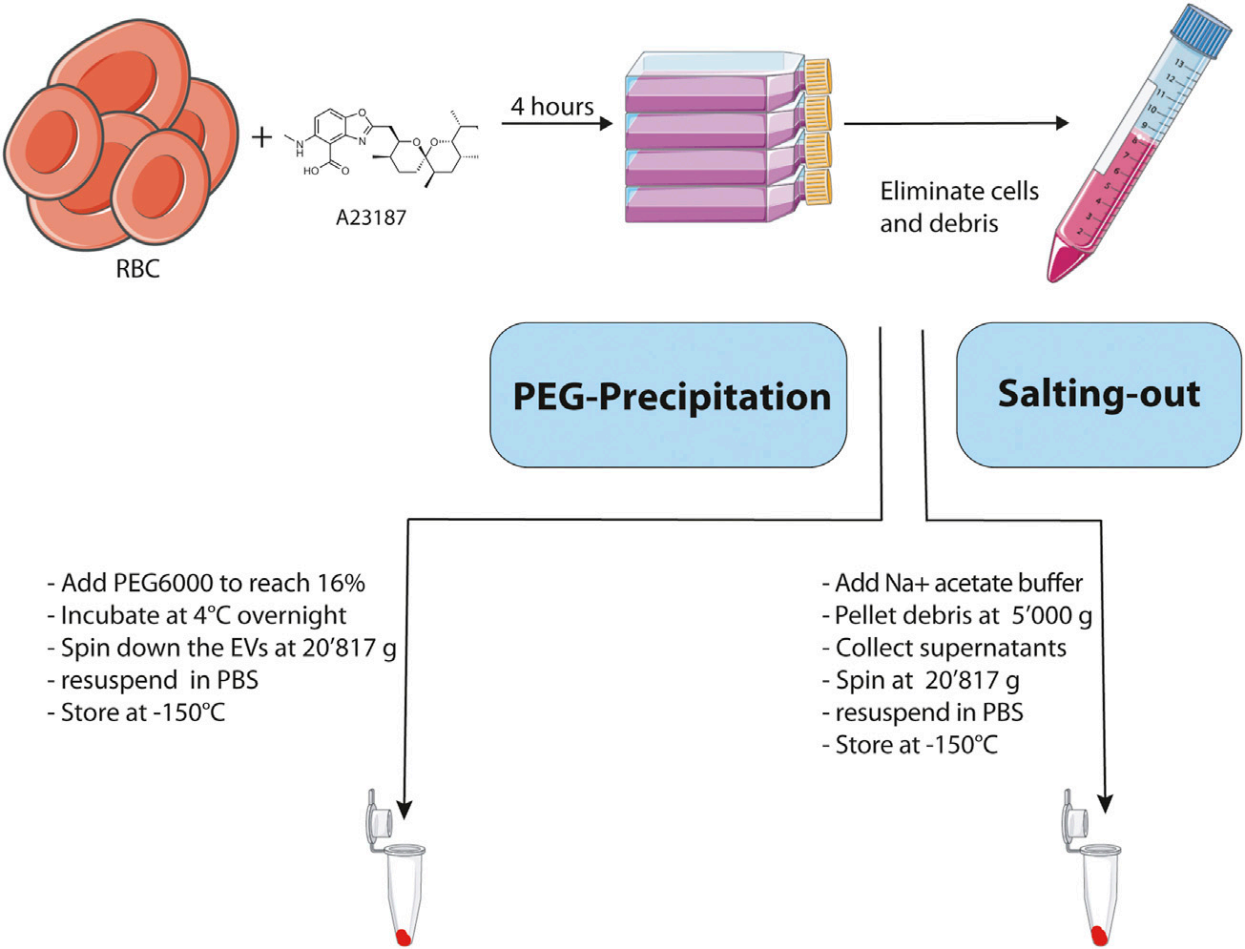
Schematic representation and description of the main steps involved in the 2 methodologies used for the isolation of EVs. Created with BioRender.com.

In order to optimize our RBC EV purification protocol, we compared 2 different approaches based on PEG precipitation and salting-out with a sodium acetate solution. The Figure 1 illustrates the strategy that we used to compare the efficiency of the two methods for enrichment of EVs derived from RBCs. First, we generated EVs *ex vivo* by incubating during 4 h freshly isolated human RBCs with a calcium ionophore (A23187, 5 μM) in HBSS buffer containing Ca^2+^ and Mg^2+^ at a hematocrit of 25%. The calcium ionophores are known to induce the release of vesicles by RBCs (Allan et al., 1976; Allan et al., 1980). After removing cells and cellular debris by differential centrifugation, the EVs were enriched from the resulting supernatants by using either a PEG solution or salting-out by sodium acetate buffer. Once the EVs were purified, we analyzed their purity and integrity by Transmission Electron Microscopy (TEM), western blot and Size-exclusion Chromatography.

### Polyethylene Glycol Treatment Results in the Enrichment of Cell Culture TEM Imaging of Healthy Human RBC EVs Extracted By PEG Precipitation

To demonstrate that PEG can be used to precipitate RBC EVs, we added PEG solutions to the A23187 induced EVs. First, the cells were pelleted at 500 g, and debris at 10′000 g the resulting supernatants were collected and a 50% PEG solution was added to reach a final concentration of 16% PEG6000. The samples were mixed thoroughly by inversion and incubated at 4°C overnight. On the next day, samples were centrifuged for 1 h at 20′817 g. The resulting pellets were resuspended in PBS and stored at −150°C.

**FIGURE 2:**
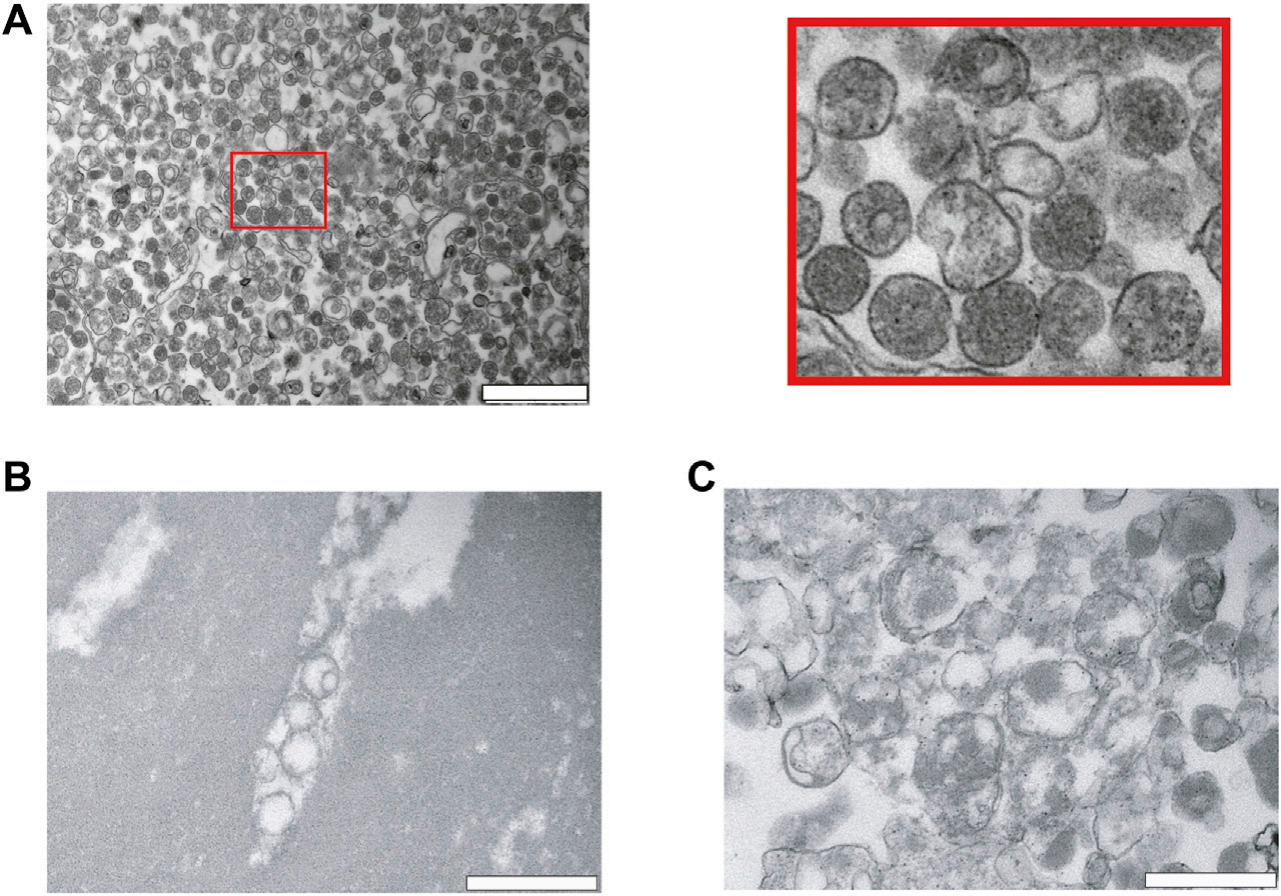
Characterization of red blood cell derived EVs isolated by PEG precipitation. **(A)** Transmission electron microscopy (TEM) visualization of RBC derived EVs isolated from A23487 treated RBCs by PEG precipitation and collected by centrifugation at 20′817 g. Representative TEM image shows individual EVs and a few clumps of varying sizes and intact lipid bilayers. The image on the right is a zoom in of the first panel. The scale bar is 1 μm. **(B)**, **(C)** Analysis of hypo-osmotic lysed RBCs by TEM. The cellular debris were pelleted by centrifugation and the pellet was resuspended in PBS for visualization by TEM. The scale bar is 1 μm.

Next, we analyzed our EV preparations by TEM, and found that PEG very efficiently precipitates and enriches for vesicles (Figure 2A). The size was relatively homogenous and varied between 100–300 nm. As expected, the vesicles have a rounded shape and are surrounded by a membrane. Most of the vesicles appeared intact and to contain hemoglobin (Figure 2A). As a comparison, we investigated lysed RBCs by osmotic pressure using a hypo-osmotic solution of salt. The RBCs lysed and released hemoglobin into the supernatant. The cellular debris were pelleted and analyzed by TEM. On the TEM images, RBC ghosts can be clearly noticed as demonstrated by large patches of membranes without a lumen. Although small vesicles are observed as well, most of them were larger in size, and they are not homogeneous in shape (Figures 2B,C).

### TEM Imaging of Healthy Human RBC EVs Extracted With Salting Out

**FIGURE 3:**
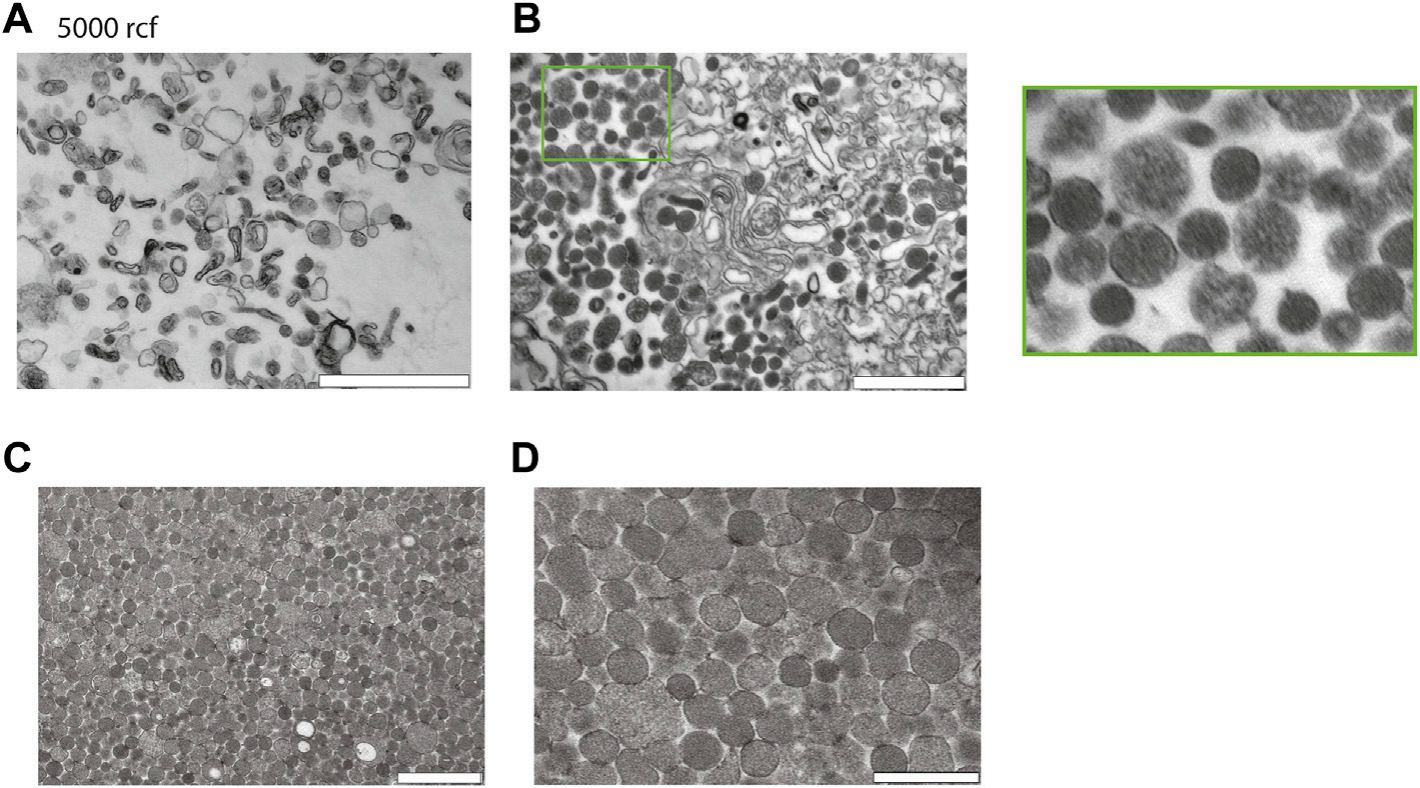
Characterization of red blood cell derived EVs isolated by salting out precipitation. **(A)** Healthy Human RBCs EVs extracted *via* Salting-out precipitation and pelleted at 5′000 g. The images were taken at 24′ × 500 magnification by TEM. Scale bar = 1 μm. **(B)** Healthy Human RBCs EVs extracted via Salting-out precipitation at 20′817 g, imaging has been taken at × 24500 magnification by TEM. Scale bar = 1 μm. **(C)** EVs were precipitated by Salting out and debris were pelleted at 5′000 g, the EVs were then collected by centrifugation at 20′817 g from the resulting supernatant. Scale bar = 1 μm. **(D)** Scale bar = 500 nm.

Next to investigate the potential of enriching EVs with salting out by sodium acetate. Intact cells and cellular debris were removed by centrifugation at 500 g and 10′000 g, respectively, and supernatants were retrieved. The cleared supernatants were then mixed with 1/10th volume of sodium acetate at a pH -4.5. The suspension immediately becomes turbid and was left on ice for 1 h with a final incubation at 37°C for 5 min. The turbid solution was centrifugated at 5′000 g and we analyzed the pellet after resuspension in PBS by TEM. The TEM revealed that salting out can precipitate vesicles as observed in Figure 3A, however after the spin at the 5′000 g, the pellet still contained a large number of cellular debris characterized by larger particles with irregular shapes. Next, to increase the collection of EVs, the precipitate was spun at 20′817 g and the pellet analyzed by TEM. As it can be observed on the Figure 3B, we recovered more vesicles, but the pellet still contained debris.

To improve the yield of EV recovery, we collected the supernatant after the spin at 5′000 g, the resulting supernatant was then spun down at 20′817 g and the pellet was analyzed by TEM. Here, the pellet contained vesicles devoid from cellular debris. The TEM revealed that EVs have a size varying from 100–300 nm. In conclusion a spin at 5′000 g is necessary to eliminate most of the debris after the salting out (Figure 3C,D).

### Salting-Out Efficiently Isolates EVs From *In Vitro* Cultures of *P. Falciparum*-Infected RBC, *P. Yoelii*-Infected Mice Plasma and Healthy Human Plasma

**FIGURE 4:**
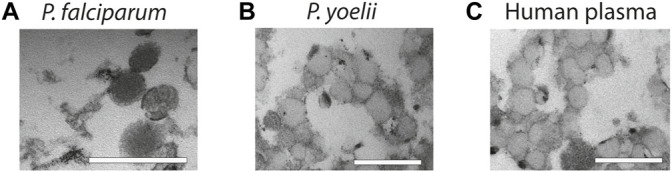
Characterization of EVs from biofluids. **(A)** EVs derived from *in vitro P. falciparum* infected RBC cultures reveal vesicular structures of 100–200 nm. Scale bar = 500 nm. **(B)** EVs isolated from plasma of *P. yoelii* infected Balb/c mice. Scale bar = 500 nm. **(C)** EVs isolated from healthy human plasma. Scale bar = 500 nm. All the EV preparations were analyzed by TEM.

Finally, to demonstrate the usefulness of our methodology with more complex samples, we used supernatants from *P. falciparum* infected RBCs, plasma from *P. yoelii* infected mice and healthy human plasma and purify them by salting out. We opted for salting out, instead of PEG precipitation because the yield is better. First, we collected supernatants from *P. falciparum* cultures and after elimination of cells and cellular debris by centrifugation, we precipitated EVs by salting out and analyzed the pellet by TEM. The TEM revealed that we were able to purify intact vesicles as it can be observed on Figure 4A. Next, we infected BALB/C mice with the *P. yoelii* rodent parasite strain and collected blood, we then isolated EVs from the plasma and again our approach was efficient at extracting EVs as observed on Figure 4B. Finally, we extracted EVs from healthy human plasma by using salting out and observed as well the high yield and specific recovery of EVs (Figure 4C).

### Size-Exclusion Chromatography Revealed a Pure Population of EVs

**FIGURE 5:**
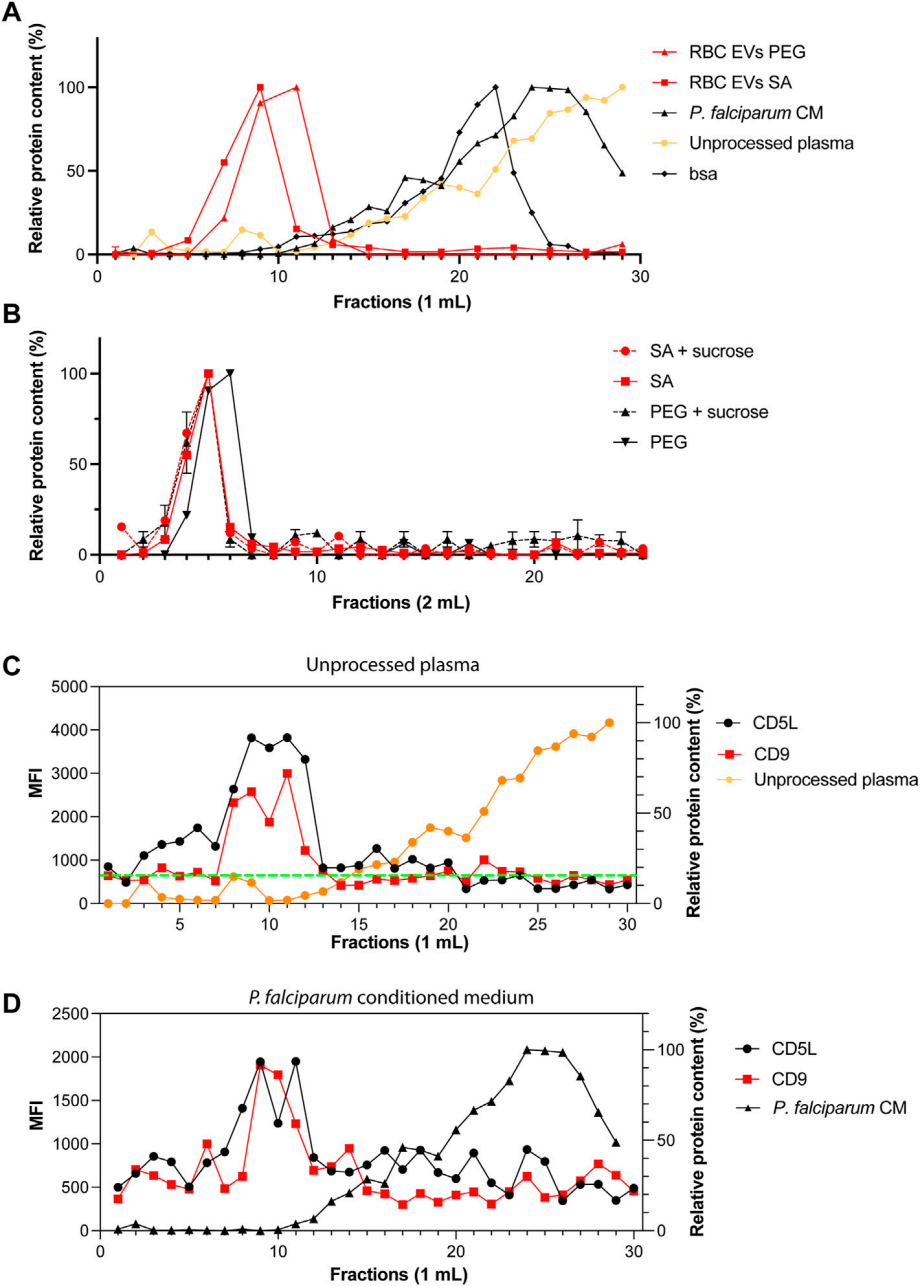
Only one population of EVs are identified by size-exclusion chromatography. **(A)** Standard curves containing the elution profiles of EVs purified by ultracentrifugation, BSA and human serum. Points represent the mean ± s.d. of two independent analyses. **(B)** Elution profile of purified RBC EVs isolated by PEG or salting out precipitation as measured by absorbance at 280 nm. The dashed lines represent EVs further purified by sucrose gradient. Points represent the mean ± s.d. of three experiments. **(C)** MFI values of CD9 and CD5L in SEC fractions. Total of 30 fractions of 1 ml were collected. **(D)** MFI values of CD9 and CD5L in SEC fractions of *P. falciparum* conditioned medium.

It is possible that we precipitated proteins and protein-complexes together with our EVs. Therefore, next, we looked at potential contaminations by using FPLCs. The fractions of 1 ml each were collected immediately after loading the column and the void volume is 4 ml. As expected, RBC EVs isolated by PEG or salting out precipitation from A23187 treated EVs, eluted in the fraction 8–11, which corresponds to the EVs profile as determined by our standard curve (Figure 5A). Only minor amounts of proteins were detected in the later fractions suggesting that most of our preparations is composed of EVs and does not contain free proteins or protein aggregates that would appear in later fractions. When fractionating unprocessed plasma, we observed a small peak of proteins in fractions 8–10, which corresponds to EVs. However, the plasma is composed mostly of proteins or protein complexes eluting in fractions 18–30. Our Bovine Serum Albumin was eluted in the fractions (12–25), as is shown on the standard curve graph. We performed a sucrose cushion in order to further eliminate protein contamination from our RBC EVs preparation. However, the sucrose cushion resulted in a significant decrease of the yield, without further improving the purity (Figure 5B). To demonstrate the presence of EVs in fractions 8–11, we performed a bead-based flow cytometry assay to detect the presence of some classical EV markers (CD9 and CD5L). We confirmed the presence of EVs in the fraction 8–11 in unprocessed plasma (Figure 5C) and *P. falciparum* conditioned medium (Figure 5D).

### Purified Vesicles Express RBC EV Markers

**FIGURE 6:**
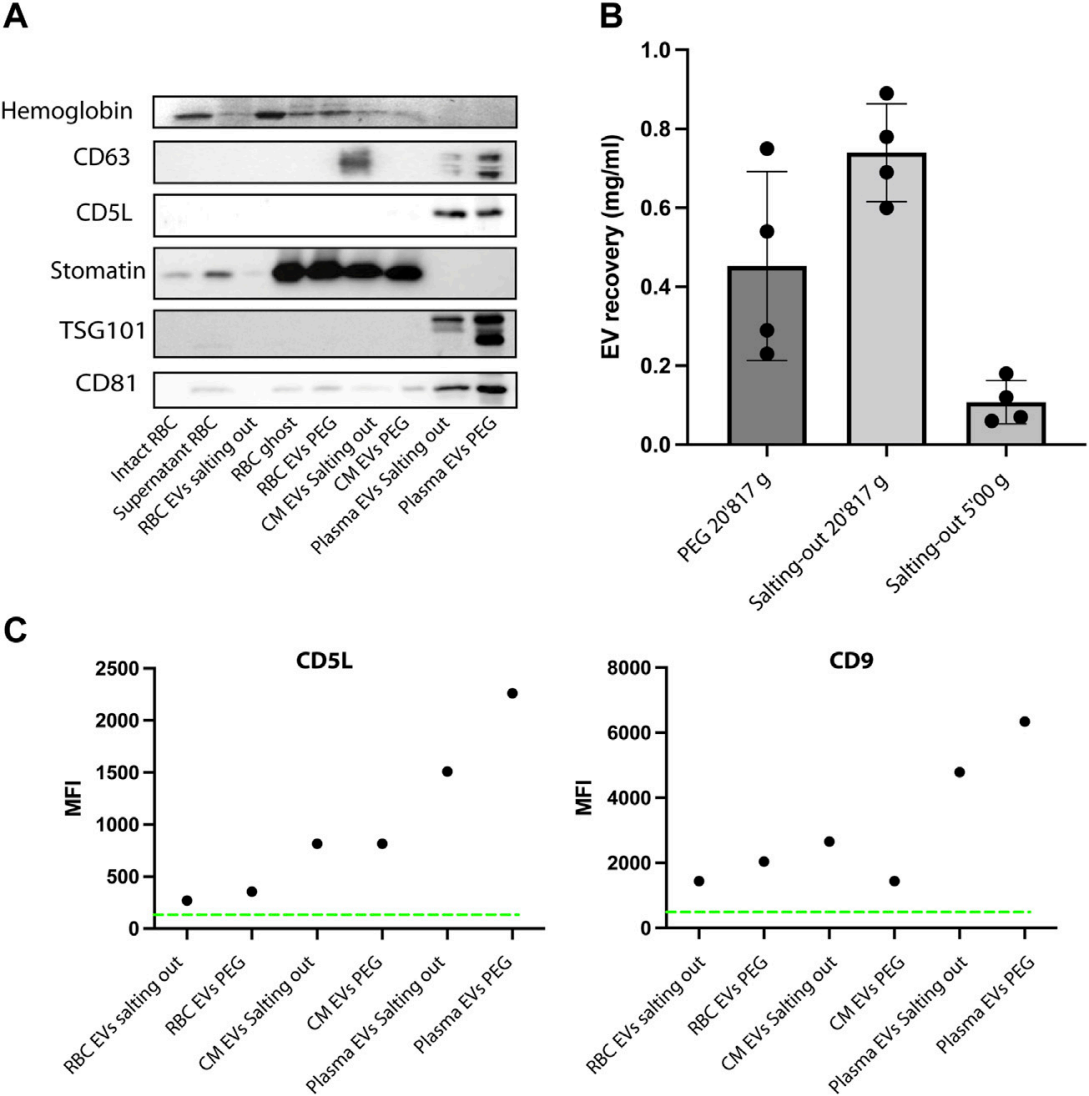
EVs express specific markers. **(A)** Western Blot analysis of RBC EV markers, stomatin, GAPDH and Hemoglobin. In total, 10 μg of protein were loaded on a SDS-PAGE gel. **(B)** A comparison of EV recovery yield between PEG and salting out precipitation as measured by protein content. The mean ± s.d. of four independent analyses is shown. **(C)** Representative experiment of purified EVs from RBCs, plasma and conditioned medium. The presence of EV markers (CD9 and CD5L), was assessed by bead-based flow cytometry assay. The Mean Fluorescence Intensity (MFI) was calculated after measuring 50,000 events (one representative of three experiments).

Next, having shown that the EVs have the expected morphology, we looked at the expression of EV markers, we used hemoglobin, CD63, CD5L, TSG101, CD81 and stomatin. Stomatin is an internal membrane protein enriched in the lipid rafts and EVs. First, as a positive control, we prepared ghosts from human RBCs (lysed RBCs) by hypotonic treatment and analyzed protein content by western blot. As expected, the stomatin protein is enriched in our ghost preparations (contain membranes), while it is absent from supernatant (cytosol). Whereas hemoglobin is present in the supernatant and absent from the ghost (Figure 6A). Hemoglobin was also detected in RBC EVs and conditioned medium (*Plasmodium falciparum* iRBCs) precipitated by PEG or salting out. Whereas it was absent from plasma derived EVs, as expected. As expected, stomatin was enriched in RBC EVs and in conditioned medium, while it was absent from plasma EVs. The tetraspin proteins (CD81, CD5L and TSG101) were detected in the plasma EVs. Next, we looked at EVs purified by PEG and salting out purification. Stomatin is present in both of our EV preparations. In addition, we could detect Hemoglobin by Coomassie staining of the SDS-PAGE gel, a result consistent with intact vesicles, containing hemoglobin in their lumen (Figure 6A). Next, we compared the yield of purification by quantifying the protein content of the EV preparations. In order to compare, we proceeded to the isolation of EVs by PEG precipitation and salting out by starting with the same amount of RBC culture. We found that Salting out is more efficient than PEG in recovering EVs. We recovered on average 0.7 mg/ml versus 0.4 mg/ml of proteins. Therefore, salting out provided a better recovery over PEG (Figure 6B). Next to test the role of Calcium in production of EVs *ex vivo* by RBCs, we pre-incubated RBCs with the calcium chelators EDTA or EGTA before stimulating vesiculation with A23187. The addition of calcium chelators diminished significantly the amount of EVs recovered by either PEG precipitation or salting out (Supplementary Figure S1). Next, the presence of classical EV markers was determined by bead-based flow cytometry on the purified EVs. The presence of CD9 and CD5L is clearly demonstrated on EVs derived by plasma, whereas the expression of those markers is lower on RBC derived EVs and *P. falciparum* conditioned medium derived EVs (Figure 6C).

## Discussion

To fully uncover the mysteries hidden behind cellular communication mediated by EVs, efficient protocols for extraction of the vesicles from cell culture supernatants, mouse blood, patient plasma have to be developed and standardize. Here we have compared two different methods to purify RBC derived EVs produced *in vitro*. Finally, we applied these methods to rodent and human plasma samples. In the first method, we used PEG precipitation (Rider et al., 2016) and in the second, salting out with sodium acetate (Brownlee et al., 2014). Both methods resulted in the efficient purification of vesicles of a size between 100–300 nm.

First, we used purified human RBCs and stimulated them with the calcium ionophore A23187, which leads to the massive release of vesicles. The vesiculation was dependent on Calcium, since the Calcium chelator EDTA or EGTA, inhibited the production of EVs.

Several approaches have been described to isolate EVs from different sources including cell culture supernatants or biofluids. Different studies have compared side by side the purification methods, there is no conclusion as to which method is more appropriate. Researchers have to consider yield, purity and cost (Sáenz-Cuesta et al., 2015).

The gold standard method for purification of EVs is differential centrifugation to eliminate cells and cellular debris (Mbagwu et al., 2017). The EVs are then pelleted from the cleared supernatants by ultracentrifugation at 100′000 g or higher speed (Andrea Hernandez-Castaneda et al., 2018). The separation principle of this method is based on the sedimentation speed difference between EVs and other particles. Some protocols add an additional step to eliminate free proteins and other debris based on density gradient by using a sucrose cushion or other density based gradient such as iodixanol (Van Deun et al., 2014). Despite being widely used ultracentrifugation has several disadvantages including co-purification of proteins aggregates and other non-EVs particles. Furthermore, ultracentrifugation might favor the aggregation of EVs, and therefore a loss of functionality (Linares et al., 2015). In addition, ultracentrifugation is time-consuming, expensive and requires the access to an ultracentrifuge. Another disadvantage is the poor scalability. In fact, working with large volume of cell culture supernatants or very small volumes from clinical samples makes it difficult to work with and might necessitate different type of ultracentrifuges. Despite those disadvantages, ultracentrifugation remains the most commonly used approach to purify EVs. Both PEG and salting out precipitation of EVs are easily scalable for small volumes such as clinical samples or for rodent experiments. Here, we were also able to precipitate EVs from large cell culture volumes up to 200 ml with minimal costs. Furthermore, there is no special instrumentation required and the time spent to manipulate the samples is relatively moderate. Both of these approaches provide an efficient way to isolate, while preserving the integrity of the vesicles. In fact, as we demonstrated by western blot, our EVs still contain hemoglobin, suggesting that they are intact.

Additional methods exist for the isolation of EVs, such as ultrafiltration (Nordin et al., 2015), fractionation, size-exclusion chromatography (Böing et al., 2014) and affinity interactions (Brennan et al., 2020), as well as microfluidic devices and microchips (Liu et al., 2017) (Shao et al., 2012). All these methods are time consuming and necessitate a specific training. Therefore, these approaches might be difficult to implement in resource limited regions.

Salting out can be performed in a few hours, whereas the PEG precipitation step is performed overnight. Both approaches can be done on small culture volume and therefore are valuable for analyzing patients biofluids that might be limited in quantity. This approach makes it also easier to work with large numbers of samples simultaneously. By adding a centrifugation step at 5′000 g to eliminate debris, we were able to improve the recovery and purity of EVs by salting out without affecting the yield. Although the mechanism responsible for EV precipitation is not fully understood, it has been suggested that the acetate-mediated removal of the EV hydration layer that promotes hydrophobic interactions result in increasing aggregation and concomitant precipitation.

The precipitated EVs can be washed to remove impurities and are readily “resolubilized” upon resuspension in acetate-free buffer at neutral pH. PEG has been used for many decades for the purification of viruses that have similar properties than EVs.

Both PEG precipitation and salting-out provided a robust highly scalable approach to purify pure EVs from *in vitro* RBC induced EVs. Therefore, these methods can be used for purification of *in vitro* generated EVs. While we used biofluids (plasma) derived from murine and human plasma the results. Although the recovery of EVs was high, the purified samples contained a significant amount of contaminations.

In conclusion, we have tested two approaches that efficiently isolate EVs from cell culture supernatants or from plasma. Both approaches do not require special equipment and therefore can be applied in resource poor regions. There is an urgent need to develop.

## Materials and Methods

We have submitted all relevant data of our experiments to EV-TRACK knowledgebase (EV-TRACK ID:EV220175).

### Cell Culture of Parasites

The *P. falciparum* strain 3D7 was used for this study. Parasites were kept in fresh type 0 + human red blood cells, suspended at 4% hematocrit in HEPES-buffered RPMI 1640 containing 10% (w/v) heat inactivated human serum, 0.5 ml Gentamycin, 2.01 g sodium bicarbonate and 0.05 g Hypoxanthine at pH 6.74. Prior to culture, the complete medium was depleted from EVs and debris by ultracentrifugation at 100′000 g for 1 h. The parasite cultures were maintained in a controlled environment at 37°C in a gassed chamber at 5% CO_2_ and 1% O_2_.

### Size Exclusion Chromatography

Experiments to determine the presence of contaminations in EVs preparations were performed essentially as described (Mantel et al., 2016). Briefly, sephacryl S-500 resin (GE Healthcare) was packed in a chromatography column (0.9 Å∼ 30 cm, 19.1 ml bed volume and void volume of 4 ml). Before injection, the column was equilibrated with 25 ml of PBS solution at 0.5 ml/min at room temperature. The column was injected with 0.5 ml of purified EVs and eluted at 4°C for approximately 1 h with PBS solution (pH 7.4) at a flow rate of 0.5 ml/min. A total of 25 fractions of 1 ml each were collected. Fractions were stored at 4°C before use. Protein molecular weight standards included BSA (67 kDa; GE Healthcare), purified EVs by ultracentrifugation and human serum.

### Bead-Based Flow Cytometry

Fractions from SEC and EV-enriched fractions from PEG and salting-out were analyzed by flow cytometry to identify the classical EV markers CD5L and CD9. As it has been described previously (Théry et al., 2006; Gualdrón-López et al., 2018). Briefly, 400 μl of each fraction was incubated with 1 μl of aldehyde/sulphate-latex beads (4 μm; 4%, ThermoFisher Scientific) by incubation for 15 min with agitation. Coupled beads were then blocked by incubation overnight with 1 ml of BCB buffer [(PBS 1X/BSA 0.1%/NaN3 0.01% (both from Sigma-Aldrich)] in a rotation device. Beads were centrifuged down at 2,000 × g for 10 min, the supernatant was discarded. The pelleted beads were resuspended in 100 μl of BCB buffer. The bead suspension was incubated with anti-CD5L antibodies (Abcam: ab45408) at 1/100 or anti-CD9 (Santa Cruz Biotechnology: ALB6) or IgG isotype control (To check) for 30 min at 4°C protected from light. After washing, samples were incubated with a rabbit or mouse secondary-antibody conjugated to Alexa 488 (both Diavona) at 1/500 dilution for 30 min at 4°C, protected from light. After two wash steps, beads were resuspended in 200 μl of PBS and 50,000 events were analyzed by flow cytometry using a BD Accuri C6 (BD Biosciences) instrument. Median Fluorescence Intensity (MFI) and count data were obtained using FlowJo v. X Software (TreeStar). As control for specificity, we have incubated SEC fraction 9 and 10 in the presence of a rabbit/mouse isotype IgG antibody and secondary-antibody Alexa (isotype control).

### Salting-Out Procedure

RBC conditioned media was cleared from cells and debris by centrifugation. 1/10th volume of Na acetate buffer (1.0 M; pH 4.75) was added to the cleared supernatant solution and incubated at 4°C under rotation. Then the solution was incubated 5 min at 37°C. The turbid suspension was centrifuged for 1 h at 5′000 g and the resulting pellet was washed with a 0.1 M Na acetate buffer solution and the supernatant was then spun at 20′817 g for 10 min. The purified EVs were resuspended in PBS and stored at −150°C.

### EVs Isolation By Polyethylene Glycol Precipitation

Vesicle-containing medium from RBC cell culture or plasma were centrifuged at 500 g for 5 min followed by 2′000 g for 30 min at 4°C to remove cells and cellular debris. After centrifugation, a 2 × PEG solution was added to an equal volume of supernatant to reach a16%- PEG concentration. The samples were mixed thoroughly by inversion and incubated at 4°C overnight. On the next day, samples were centrifuged in a tabletop centrifuge at 20′817 g for 1 h at 4°C. conical tubes were then decanted, and allowed to drain for 5 minutes, tapping occasionally to remove excess PEG. The resulting pellet was suspended in 50–500 μl of particle-free PBS (pH 7.4). subsets of samples were then either stored at -150°C or used straight away for experiments.

### Transmission Electron Microscopy

A total of 10 μg of EVs purified by Salting out and PEG were fixed in 2% PFA/2.5% GA (EM grade) in 0.1 M Cacodylate Na Buffer, postfixed with an aqueous solution [1% OsO4 and 1.5% K_4_Fe (CN)_6_], and embedded into epon. Ultrathin sections (50 nm) were contrasted with lead citrate and uranyl acetate and analyzed with a CM 100 (Philips).

### Western Blotting

Samples were collected and purified as described in each specific experiment. For SDS–polyacrylamide gel electrophoresis the pellet was washed three times in PBS and taken up in reducing SDS sample buffer (Invitrogen, Carlsbad, CA). Proteins were separated on 4–12% Bis-Tris gels (Invitrogen) and proteins transferred onto Immun-Blot PVDF membranes (Biorad), according to standard protocols. Antibodies used are anti-stomatin (clone M-14; Santa Cruz Biotechnologies), CD63 (clone E-12: Santa Cruz Biotechnology), CD81 (clone 5A6; Santa Cruz Biotechnology), CD9 (ALB6; Santa Cruz Biotechnology), TSG101 (4A10; Novus Biologicals) and CD5L (ab45408, Abcam). Secondary antibodies (IR-Dye-conjugated) were goat anti-rabbit and goat anti-mouse immunoglobulin (LICOR, Lincoln, NE). Immunoreactive bands were detected using the Odyssey imaging system (LICOR).

### Red Blood Cells Isolation and Treatment With Calcium Ionophore

Venous blood, collected in acid-citrate-dextrose, was obtained from healthy adult volunteers. The RBCs were washed three times with PBS. And stored at 4°C at a hematocrit of 50%.

Treatment with calcium ionophore. Ca^2+^ionophore treatment was performed at 37°C by addition of A23187 (Sigma-Aldrich) at a concentration of 5 μM in a HBSS buffer containing Ca2+. Unless otherwise indicated, incubation time of RBCs with ionophore was 4 h.

## Data Availability

The raw data supporting the conclusions of this article will be made available by the authors, without undue reservation.
