# Supplementary figures and images for: Validation of Effective Extracellular Vesicles Isolation Methods Adapted to Field Studies in Malaria Endemic Regions
Source: Front Cell Dev Biol. 2022 May 16;10:812244. doi: 10.3389/fcell.2022.812244 (PMC9149222; doi:10.3389/fcell.2022.812244)

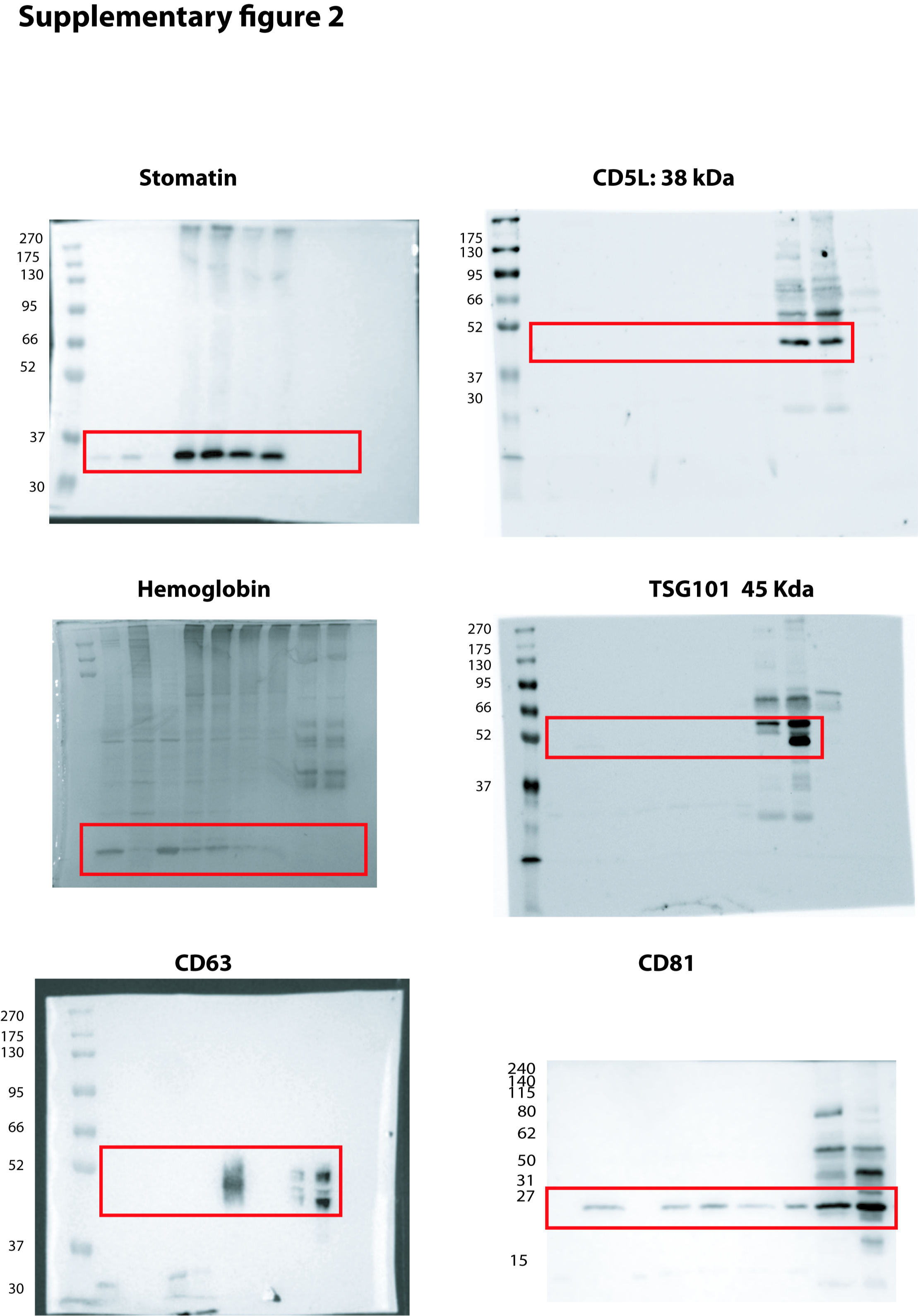

Supplement: Supplementary file 1 [file Figure8.TIF]

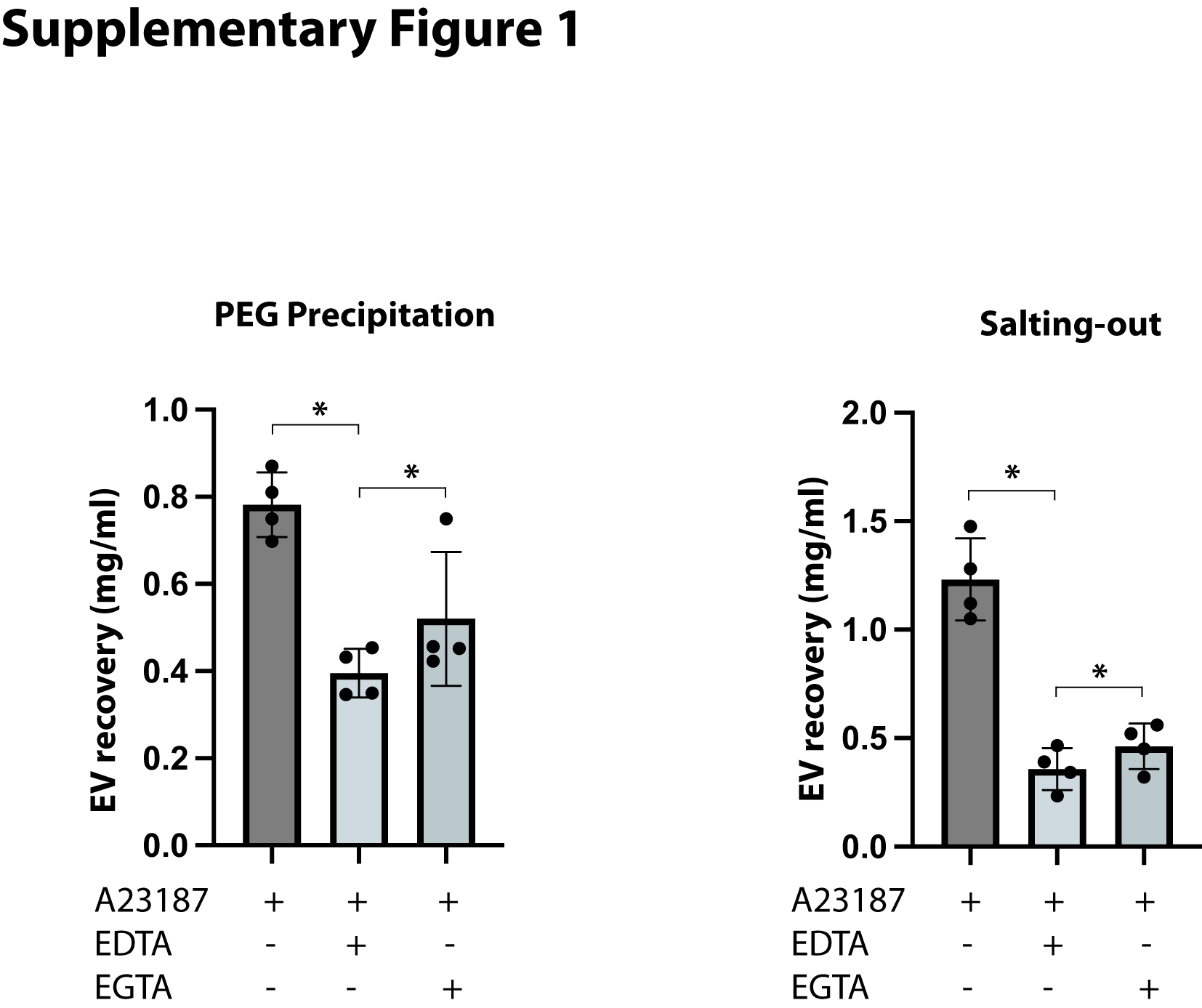

Supplement: Supplementary file 2 [file Figure7.TIF]
